# Women’s Experiences of Vulvodynia: An Interpretative Phenomenological Analysis of the Journey Toward Diagnosis

**DOI:** 10.1007/s10508-018-1246-z

**Published:** 2018-07-25

**Authors:** Rebekah Shallcross, Joanne M. Dickson, David Nunns, Kate Taylor, Gundi Kiemle

**Affiliations:** 1 Doctorate in Clinical Psychology, The University of Liverpool, Liverpool, L69 3GB UK; 2 Centre for Academic Primary Care, Population Health Sciences, Bristol, The University of Bristol, Bristol, UK; 3 Department of Psychological Sciences, University of Liverpool, Liverpool, UK; 4 Department of Psychology, Edith Cowan University, Joondalup, Australia; 5 Department of Gynaecological Oncology, Nottingham University Hospitals, Hucknall Road, Nottingham, Nottinghamshire, UK; 6 Vulval Pain Society, PO Box 7804, Nottingham, UK

**Keywords:** Vulvodynia, Vulval/vulvar pain, Interpretative phenomenological analysis

## Abstract

Vulvodynia is the experience of idiopathic pain characterized by burning, soreness, or throbbing in the external female genitalia or vulva and is estimated to be experienced by 4–16% of the female population, yet only half of women seek help regarding their symptoms. Of the women who do seek help, only around 2% obtain a diagnosis. Therefore, the aim of the current study was to explore the experiences of women with vulvodynia on their journey toward diagnosis, by using semi-structured interviews and an interpretative phenomenological analysis (IPA) methodology. Eight women were interviewed, and their experiences were analyzed and interpreted into three master themes, each with constituent sub-themes: (1) The Journey Is a Battle, (2) “What Is Vulvodynia?”: Ambivalence Toward Diagnosis, and (3) Patriarchy, Women, and Sex. Overall, women perceived a healthcare system which was dismissive and shaming, with an inadequate knowledge of vulvodynia. This in turn impacted on women’s psychological well-being. Psychological understanding, one-to-one therapy, and consultation and training for healthcare professionals may help to improve the psychological well-being of women with vulvodynia.

## Introduction

Vulvodynia is the experience of idiopathic pain characterized by burning, soreness, or throbbing in the external female genitalia or vulva (Nunns & Murphy, 2012), and is experienced by 4–16% of the female population in the U.S. (Eppsteiner, Boardman, & Stockdale, 2014), with no UK population estimates available. Research has explored several causative factors including neuropathic pain, psychosocial influences, and infection; however, the etiology remains unknown, and successful therapy often involves a multidisciplinary approach (Eppsteiner et al., 2014). The subjective impact of vulvodynia is idiosyncratic, but common difficulties include using tampons, sitting, wearing jeans, tights, or trousers, engaging in penetrative sex, or exercising, which in turn impact on women’s day-to-day functioning, including both employment, leisure, and caring activities. Vulvodynia also impacts on intimate relationships and psychological well-being, including increases in depression, anxiety, and lowered self-esteem (Gates, 2001; Gates & Galask, 2001; Sackett, Gates, Heckman-Stone, Kobus, & Galask, 2001). Social constructions around sex and womanhood also exacerbate psychological difficulties by increasing shame, silencing, and guilt at not being able to “perform as a woman,” which in turn leads women to feel de-gendered and no longer “a real woman” (Ayling & Ussher, 2008; Kaler, 2006; Marriott & Thompson, 2008). As such, the psychological impact of vulvodynia should be understood in the context of individual experiences, but also within a societal context, and experiencing pain in an area intrinsically linked with sex, gender, and femininity (Shallcross, Dickson, Nunns, Mackenzie, & Kiemle, 2018).

Research focused on women’s healthcare experiences suggests that around 35% of women in the U.S. attend more than 15 appointments and wait more than 36 months between the onset of symptoms and receiving a diagnosis of vulvodynia (Connor, Brix, & Trudeau-Hern, 2013). While only half of women experiencing symptoms diagnostic of vulvodynia seek help, even fewer (less than 2%) obtain a diagnosis (Reed et al., 2012). The reasons for this are unknown. Several qualitative papers focusing on the impact upon intimate relationships and sexual functioning (Ayling & Ussher, 2008; Kaler, 2006; Marriott & Thompson, 2008), or the effectiveness of interventional multidisciplinary groups (Brotto, Basson, Carlson, & Zhu, 2013; Munday, Buchan, Ravenhill, Wiggs, & Brooks, 2007; Sadownik, Seal, & Brotto 2012a), provide some suggestions such as side effects of medication (Munday et al., 2007), delays in treatment (Buchan, Munday, Ravenhill, Wiggs, & Brooks, 2007), and the implication that the pain was “all in their head” (Brotto et al., 2013; Kaler, 2006; Marriott & Thompson, 2008; Sadownik, Seal, & Brotto, 2012b) as possible reasons women do not seek help/receive a diagnosis. However, these papers do not specifically explore women’s journeys toward diagnosis—their findings are secondary to their primary exploration of intimate relationships or intervention effectiveness. One study that did specifically focus on the “journey into treatment” concluded that “vulvodynia is poorly recognized, and the delay to diagnosis adversely affects patients, exacerbating the severity of their symptoms” (Buchan et al., 2007 p. 15). However, this specific research had several flaws, particularly in relation to data analysis which was not adequately described within the text, making it difficult to ascertain exactly how data were analyzed, and how themes were arrived at. Similarly, no information pertaining to the epistemological or ontological standpoint of the authors is described. Buchan et al. did not adequately describe the relationship between the researcher(s) and the participants, nor did they critically examine their own role, potential bias and influence throughout the research process. Finally, their findings are not discussed in relation to strengths and limitations, nor in relation to previous literature, both for and against, which makes credibility and applicability of the research hard to establish. Overall, the analysis is thematic and descriptive, with a lack of discussion regarding both convergent and divergent themes, which means that the reader gets a sense of the practical barriers women with vulvodynia may face, but not an in-depth analysis of how women *experienced* their journeys. Therefore, the current study aims to ask women, using in-depth interviews, about their journey toward obtaining a diagnosis and what this experience was like for them, in order to understand the barriers in obtaining a diagnosis of vulvodynia, despite women experiencing symptoms consistent with this diagnosis (Reed et al., 2012). It will address the limitations of the previous work on the journey-to-diagnosis by using an appropriately in-depth methodology (IPA) in order to address the research question, by adequately describing and critiquing the relationship between the researcher and the research, as well as examining the epistemological and ontological standpoint of the research and the potential bias brought by the researcher (see section: IPA, Reflexivity, and Validity), and finally by providing in-depth description of the analytic process (exploring both convergent and divergent themes) in order to understand women’s experiences.

Before addressing this question specifically, it is helpful to explore previous feminist literature which helps to provide a contextual backdrop to the experiences of women in the healthcare system. Although not specific to vulvodynia, this provides some interesting insights, suggesting what women with vulvodynia may experience, and situating our findings in relation to these previous findings (Du Plessis, 2015; Exner, Dworkin, Hoffman, & Ehrhardt, 2003; Krieger & Fee, 1994; Maines, 1999; Marken, 1996; Martin, 1987; McPhillips, Braun, & Gavey, 2001; Springer, Stellman, & Jordan-Young, 2012; Stacey & Olesen 1993; Tiefer, 2001). Although progressively changing, research and medicine largely remain male-dominated (Stacey & Olesen, 1993). Specifically, this literature documents the historical and present-day limitations of research into issues affecting women, and the problems with clinical healthcare practice characterized by patriarchal and androcentric beliefs and assumptions, often leading to the negation of women’s experiences.

Historically, research seems to have been heavily influenced by sociopolitical contexts. For example, research concluding that women were a hindrance in the workplace due to menstruation, was popular around the end of World War I when men returned, wanting to take back the waged work women had been doing in their absence. Contrary research produced at the start of World War II, when women were needed to move back into the labor market, concluded that menstruation was no longer a liability in the workplace (Martin, 1987). In addition to the influence of the sociopolitical context, the perspective from which research is approached is also of the utmost importance. For example, seeing women’s premenstrual experiences as capacities and gains (such as increased capacities in creativity and sensitivity) rather than problems and liabilities, changes the interpretation of Premenstrual Syndrome (PMS) and its impact on women in the workplace (Martin, 1987). Unhelpful sociopolitical perspectives continue to be reflected in modern research, particularly when it comes to sex hormone or sex chromosome research, which often reflects a biological-determinist view, examining “sex” as if it were a biological mechanism per se, while ignoring the impact of “gender” (and gendered experiences such as inequality, poverty, lack of power) on health outcomes. Indeed, according to Krieger and Fee (1994), these biological-determinist views have become fundamental parts not only of the research agenda, but also of the medical curriculum and practice. Springer et al. (2012) argue that as sex and gender are inextricably linked, we should be examining outcomes through an interactive biosocial lens of “sex/gender,” which would greatly further our understanding of health-related research questions. Despite this landscape, gender is gradually emerging as a critical variable in outcomes in Western studies of illness. Research should continue to emphasize the effects of gender (as well as race, ethnicity, and class) and their relationship to health, in order to provide comprehensive (not just biological) understandings of the complexities of women’s health (Stacey & Olesen, 1993).

As with research, clinical practice also suffers from androcentric beliefs and assumptions. Historically, women’s health has been predominantly concerned with women as wives and mothers, with women’s needs assumed to be met by maternal and child health programs, with less attention paid to women’s non-reproductive health (Krieger & Fee, 1994). Feminist sexology literature reveals that the medical profession has been characterized by phallocentrism and constructed within androcentric views of sexuality (Maines, 1999), particularly in regard to sex whereby “real” sex equals penetration of the vagina by the penis (coitus), placing this particular sexual act as central to “normal” heterosex (McPhillips et al., 2001; Maines, 1999), as well viewing penile erections as the essence of male sexuality and satisfaction, and the expectation of female submission to provide pleasure and meet the sexual as well as the emotional needs of men (Du Plessis, 2015). As such, feminist sexology literature demonstrates how these factors impact upon women’s experience of sexuality by restricting women’s sexuality to a framework that is inflexible and limited in possibilities to penetration.

Furthermore, clinical practice is historically rooted in male perspectives and understandings (Exner et al., 2003), therefore negating women’s experiences and their needs. Literature suggests that doctors may perceive female patients as “inherently dependent” and “lacking in common sense,” a view that rationalizes “paternalistic attitudes and advice” (Gannon, 1998, p. 295). Similar research highlights a male bias in medicine, whereby the unchanging male body that maintains a state of equilibrium and stability is seen as “normal,” while the constantly changing female body (through menstruation, pregnancy, and menopause) is seen as pathological (Marken, 1996), with the problems experienced by men being caused by outside circumstances and other people (women), while the problems of women are caused by their own internal failure; a biological “malfunction” (Martin, 1987).

In particular, Martin (1987) talks about the bind that women find themselves in when it comes to premenstrual experiences; on the one hand, the medical diagnosis of PMS or premenstrual tension (PMT) gives legitimacy to their experiences as “real” and not “all in their heads,” but on the other hand these diagnoses may lend themselves as “proof” that women are “moody” and “less productive” when menstruating (Martin, 1987; Marken, 1996; Tasca, Rapetti, Carta, & Fadda, 2012). For example, women’s experiences of anger and rage as part of PMS often lack any consideration of why women might feel extreme rage at a time when their usual emotional control is reduced (Martin, 1987). Often a diagnosis of PMS assumes a malfunction or deficiency within the woman, while societal oppression, lower wage scales, fewer opportunities for professional advancement, and expectations of taking on roles that require nurturance and self-sacrifice (among others) are often overlooked in clinical practice (Martins, 1987).

Both research and clinical practice would benefit from understanding what is similar and what is different between men and women, examining and accounting for social divides across gender, race/ethnicity, and social class. Assumptions that examining “sex” alone as a variable that will provide answers must change. Instead, it is imperative to examine the context of health experiences within a sex/gender framework and begin to challenge some of the norms and assumptions within research and clinical practice that continue to create health inequalities (Krieger & Fee, 1994). In particular, the theme of the medical profession viewing women through a paternalistic lens and as pathological by nature, while ignoring social structures that negatively impact upon women, should be borne in mind when considering the experiences of women with vulvodynia (Shallcross et al., 2018).

In summary, previous research fails to offer sufficiently detailed, explanative, and robust insights into how women experience their journey toward diagnosis within the healthcare system, and how this impacts upon their pain, functioning, and well-being.

Therefore, the current paper aims to explore critically and in depth the experiences of women diagnosed with vulvodynia within the UK healthcare system, specifically their journey toward diagnosis, while seeking to address the methodological issues of previous research. To do this, qualitative methodology and IPA (Smith, Flowers, & Larkin, 2009) was used, as a way of getting close to the participants’ personal worlds, while recognizing that meanings are constructed within a social context.

## Method

The study was developed with members of the London Vulval Pain Support Group (a patient support group), and the Vulval Pain Society (VPS, a national UK charity and patient support group), whose members identified their experiences of the journey toward diagnosis as a significant area in need of further research. Support and advice was sought throughout the research process from an expert by experience and service user. Ethical approval was obtained from a University Research Ethics Committee.

### Participants

In keeping with IPA methodology, the study aimed to recruit between four and ten participants, allowing for detailed analysis of individual accounts as well as the development of meaningful cross-case analysis for patterns of similarity and difference (Smith et al., 2009). Women living in the North West of England, who were also supporters of the VPS were invited to take part in the study via VPS Web site advertisements and email lists. Inclusion criteria were: women with a medical diagnosis of vulvodynia (diagnosed by a qualified medical practitioner), over 18 years of age, and able to speak and understand sufficient English to take part in the interview. In discussion with an expert by experience from the VPS, the criterion of receiving a diagnosis no less than 6 months ago and no more than 7 years ago was also added in order that participants had had enough time to reflect on their experiences of the journey toward diagnosis, but not so long ago as to hinder recall. Eligible women wishing to participate were sent the study information sheet, following which an interview was arranged. Informed consent in writing was obtained from all individual participants included in the study.

### Procedure and Measure

The interview was conducted in a flexible manner, using a semi-structured schedule designed to facilitate an enabling interview for participants to tell the story of their experience. The aim of the semi-structured interview schedule was to use open-ended and non-directive questions to facilitate in-depth and at-length dialogue on the part of the participant (Smith et al., 2009). Questions (and possible prompts) were developed that aimed to suspend, as far as is possible, prior assumptions about the participants and their experiences, covering the following areas: onset of symptoms (e.g., What symptoms did you experience?), contact with services (e.g., “How did you experience your initial contact with services?”; “What happened next?”), and the role of diagnosis (e.g., “What has been your experience of having a diagnosis?”). The mean length of the interviews was 78 min (range 53–109 min). Interviews were transcribed, and all identifiable information was removed from transcriptions, with pseudonyms used throughout. Qualitative data analysis software QSR-NVIVO 10 (QSR International Ltd., 2012) was used to aid storage and retrieval of the data.

#### IPA, Reflexivity, and Validity

IPA (Smith, 1996; Smith et al., 2009) has been widely used in health research, including research on vulvodynia (Marriott & Thompson, 2008). It aims “to understand how people make sense of events, relationships, and processes in the context of their particular lifeworlds” (Larkin, Eatough, & Osborn 2011, p. 330). It is influenced by the philosophical underpinnings of phenomenology, hermeneutics, and idiography.^1^

IPA is phenomenological in its detailed examination of the personal, lived experience of participants, exploring how participants make sense of these experiences (Smith, 2004). IPA methodology aims to get close to the participant’s personal world (Smith, 2007), as far as is possible, by adopting an “insider perspective” (Conrad, 1987; Smith, 1996). However, IPA recognizes that this is not directly and completely possible, as access to the participant’s personal world, as it is complicated by the researcher’s own conceptions (Smith, 1996). As such, IPA recognizes that the research process is dynamic; acknowledging the “double hermeneutic” or “two-stage interpretation process” whereby the participant seeks to make sense of their world, and the researcher (rather than multiple researchers and coders) seeks to make sense of the participant’s sense-making (Smith, 2007).

Moreover, IPA is idiographic, acknowledging that individual interpretation takes place within the context in which the phenomenon transpires (Larkin, Watts, & Clifton, 2006) and is informed by the theoretical perspective of “symbolic interactionism” (Smith, 1996): how meanings are constructed by individuals within both a social and personal world (Smith, 1996). As such, IPA takes account of individual differences in meaning-making within a social context and thus is well suited to studying the intimate personal nature of the experience of vulvodynia within the context of the healthcare system, itself set within a wider social context.

Guidelines for the process of analysis were followed (Smith et al., 2009). Each transcript was analyzed individually before moving to the next case. Each transcript was read, re-read, and highlighted for phrases and paragraphs of particular interest. The transcript was then subjected to a detailed analytical reading while also coding the text at descriptive, linguistic, and conceptual levels in the right-hand margin. Following this, the initial coding was developed into emergent themes which were recorded in the left-hand margin. The emergent themes were then scrutinized and clustered to create subordinate themes, in turn clustered into superordinate themes. This procedure was then completed with all subsequent transcripts. Following the analysis of individual transcripts, the tables of super and subordinate themes were examined to detect patterns across cases, with attention paid to both convergent and divergent themes, in order to identify higher-order concepts, resulting in a table of key master themes, with sub-themes nested within each (see Table 2). The researcher moved between the individual (idiosyncratic) level, staying close to the data, developing interpretations at different levels (social comparison, metaphor, and micro-analysis), as well as adopting a theoretical stance, taking an overview of the data, refining themes at every level throughout the analytic process using abstraction, subsumption, polarization, and contextualization as ways of looking for patterns and connections both within and across cases and thus continuing the hermeneutic circle. As such, the analysis was not solely concerned with moving from the individual (raw data) to the whole (a master table of themes), but rather through analysis, and the writing process, there was opportunity to move in the other direction also. For example, having analyzed all the transcripts, the researcher focused the analysis to include a deeper and more detailed reading of particularly resonant passages, which then informed and enlightened the whole analysis, and so on, thus moving the analysis to a deeper level of interpretation (Smith et al., 2009). As is consistent with the iterative and dynamic nature of IPA, it is important to note that analysis did not stop here, but rather continued to develop during the writing up of themes, illuminating, strengthening, and thickening the narrative emerging from the analysis. Furthermore, drafting and re-drafting also allowed the author to become clearer in, and deepen, her analysis and argument (Smith et al., 2009). In keeping with IPA methodology, the research team met for discussions regarding the lead author’s analysis throughout this process, and a reflexive diary was utilized in order to recognize the potential biases of the lead researcher, to acknowledge and curtail their impact upon the research process, and to ensure the validity and quality of the research.

The lead author (RS) conducted the analysis. She is a 30-year-old, female psychologist of white, British background. Her previous and current research is in women’s health and mental health and she has worked clinically within psychosexual health services. The author identifies as a feminist and as such believes that both men and women suffer the repercussions of patriarchal and misogynistic attitudes that are subtly and sometimes not-so-subtly ingrained in society. The author’s reading of feminist sexology literature (Moynihan, 2003; Tiefer, 2000, 2001) has informed her thinking about sexuality, specifically around the medicalization of women’s sexuality, which promotes a universally generic, function-focused and heteronormative sexuality, ignoring the areas of sexuality that women find most distressing, such as loss of intimacy and inability to fulfill the desire to please their partners. The author believes that professionals should look to non-medical frameworks, which have been promoted by academic, feminist, gay/lesbian, and political writers (Tiefer, 2001). The author does not have first-hand experience of idiopathic vulval pain; however, she has been allowed an insight into the experience of vulvodynia through a close friend who shared some of her experiences with the researcher prior to the commencement of this research, and throughout the research process. The author rejects both the extreme positions of naive realism (or positivism) and extreme relativism (or radical constructionism) and adopts a critical realist epistemological and ontological position, in keeping with where IPA is commonly positioned (Bailey, 2011; Nightingale & Cromby, 1999).

## Results

Twenty women contacted the researcher. Of these, two women subsequently decided not to go ahead with the study and two women were unobtainable following the initial contact. Six women were not eligible to participate (no formal diagnosis (*n* = 1), living outside of recruitment area (*n* = 1), diagnosed more than 7 years ago (*n* = 4)), and two women contacted the researcher after recruitment was closed on the VPS Web site. Therefore, eight women, aged between 23 and 70 years, who met all the inclusion criteria, were interviewed (five in their own homes and three at a University site). Table 1 outlines the demographic information of all included participants.

**Table 1:** Demographic information of participants

	Lilly	Amy	Vicky	Laura	Clara	Liz	Bessie	Josephine
Age	33	24	48	23	57	58	67	70
Other Relevant Diagnosis	MRI revealed some scar tissue	Spastic paraplegia	Lichen Sclerosus	Vulval eczema	Urethral syndrome and fissures	N/A	Varicose vein	Pudendal neuropathy
Relationship Status	Single	Boyfriend	Complicated	Recently Single	In a relationship	Married	Married	Married

Three master themes with constituent sub-themes were developed, as outlined in Table 2.

**Table 2:** Table of master themes and sub-themes and recurrence across participants

	Lilly	Amy	Vicky	Laura	Clara	Liz	Bessie	Josephine
* The Journey is a Battle*	YES	YES	YES	YES	YES	YES	YES	YES
On a Journey with No Direction	YES	YES	YES	YES	YES	YES	YES	YES
The Status and Power of the Medical Model	YES	YES	YES	YES	YES	YES	YES	YES
The Psychosocial Impact of the Journey	YES	YES	YES	YES	YES	YES	YES	YES
*“What is Vulvodynia?”: Ambivalence Toward Diagnosis*	YES	YES	YES	YES	YES	YES	YES	YES
“Vulvodynia is a Bit of a Cop-Out”: Limitations of a Diagnosis	YES	NO	YES	NO	YES	YES	YES	YES
The Value of Diagnosis	NO	YES	YES	YES	YES	YES	NO	YES
*Patriarchy, Women, and Sex*	YES	YES	YES	YES	YES	YES	YES	YES
Narratives Around Womanhood: A Barrier on the Journey	NO	YES	YES	NO	YES	YES	YES	NO
Female Sexuality, Shame, and Stigma	YES	YES	YES	YES	YES	YES	NO	YES
“The Old Boys’ Brigade” and The Medical Profession	YES	YES	YES	NO	YES	YES	YES	YES

KEY: Sub-themes; master themes

### Master Theme 1: The Journey Is a Battle

You’ve got to fight…mmm, yes, so if you want me to fight, standing up for vulvodynia, I’m here (Josephine).

Josephine’s description of her journey as a “fight” was echoed by several women, for example “I fought for 3 years” (Amy); “It’s so hard trying to fight for yourself” (Liz); “It’s this constant battle of feeling believed” (Laura). Metaphorical language such as “It gives you very conflicted feelings” (Lilly) suggests an inner state of conflict created by battling or fighting. The metaphor of a battle, fight, or conflict conveys a journey toward diagnosis that is long, hard, laborious, harmful, and traumatic, both physically and psychologically. The following three sub-themes highlight particular areas that contributed to the overall sense of the journey as a battle.

### Sub-theme 1: On a Journey with No Direction

Participants often reported a sense that “GPs haven’t got a clue” (Amy) or “can’t/don’t understand” (Josephine/Bessie), with the majority of women describing how this left them feeling lost (“who do you turn to?” Liz), “dismissed” (Clara), or that they should “just get on with it” (Liz). GPs’ limited knowledge, preventing them from offering proactive help, was the first barrier women reported being faced with on their journeys. Poor medical knowledge surrounding vulvodynia led to women being inappropriately referred, wrongly diagnosed, and prescribed iatrogenic or unhelpful medication, such as repeated prescriptions for vaginal Candida (antifungal cream) which “actually makes the skin around your vulva worse” (Amy), thus contributing to the exacerbation of vulvodynia.

In turn, women reported that lack of GPs’ knowledge necessitated them researching the internet for information on vulvodynia (Bessie; Vicky; Liz), support networks (Clara; Vicky), appropriate clinics to request referrals to (Clara; Bessie), different interventions to try (Liz; Bessie), or different “tests” to have done (Vicky), further highlighting the knowledge gap regarding services and treatment. Reported barriers that prevented navigation of the “fragmented system” (Vicky) included: lack of continuity of care, a need to chase referrals, a lack of ongoing support, long waiting times to be seen by specialists once a referral had been secured (often between 6 and 18 months), a feeling of being “in the dark” (Liz), and a sense of going “back and forth” (Amy):

It’s just, it’s horrible, it’s horrible, the amount of times that I’ve just been shooed off, and being passed from pillar to post and erm, I don’t, I don’t really know how to explain it, erm, I’ve just got this, this massive fear that, of being unbelieved (Laura).

Laura’s use of the word “shooed” brings to mind a small animal or child, who is being frightened away and forced to leave. It conveys a sense that Laura feels of being experienced as a nuisance by doctors; she feels forced out and perhaps frightened to return to ask again for help. The phrase “passed from pillar to post” is thought to derive from punishment (the flogging post and the pillory), a punishment designed to cause both pain and public humiliation. Laura may therefore be understood to feel like a nuisance, to experience a sense of being punished and being forced to remain in pain through being dismissed. Finally, Laura uses the present tense to describe her “fear” of being “unbelieved,” creating a sense that her “battle” to obtain a diagnosis has left her still currently experiencing fear and anxiety. Much in the same way that a battle might leave lasting “scars,” Laura may feel that at any moment, the belief and validation acquired through diagnosis could be taken away: she may have to return to being no longer believed—she may have to return to the battle.

Overall, the experience of the journey within the healthcare system left six women (Lilly, Amy, Laura, Liz, Bessie, and Josephine) considering treatment privately, in order to gain a sense of direction and help (access adequate knowledge to diagnose and treat vulvodynia in an acceptable time frame). As Bessie states “I wouldn’t hesitate, that’s the only thing I would say: pay for it.”

### Sub-theme 2: The Status and Power of the Medical Model

They can be very domineering, can’t these consultants, think they’re gods (Josephine).

Josephine uses the word “domineering,” conveying a sense of feeling subjugated and oppressed. The use of the word “gods” evidences Josephine’s belief that the consultants consider themselves “god-like,” with ultimate power over “life and death” (Josephine), illustrating the enormity of a power imbalance experienced by Josephine.

This perception of a power imbalance was echoed by other women, who reported instances where their knowledge and expertise were undermined and ignored by silencing actions. For example, Vicky had her hands “slapped away” by a professional, when trying to show them where the pain was. Similarly, Liz experienced “you’ve come to see me to find out what’s the matter with you and you’ve come and told me what’s the matter with you” from her consultant, when she showed him a booklet on vulvodynia. This suggests that perhaps this particular physician wanted to retain their power of knowledge and therefore was not open to the research Liz had done, despite an incorrect diagnosis and unhelpful prescribing. The perceived need for power to be maintained by the professionals in these instances hindered the progress of both Vicky’s and Liz’s journey.

Three participants (Laura, Lilly, Clara) reported instances whereby they felt violated by vulvovaginal examinations that produced significant psychological distress: “it actually felt like I’d been assaulted…I felt really violated” (Lilly). At the age of 23, Laura had counted over 50 doctors that had examined her. Here she recounts one such examination:

and he was like, right I want to examine you, so it was a case of right, OK, you know, and so he tried to examine me, bearing in mind that he’s a urologist, so why, why would he need to do anything with my vagina? without warning me, bearing in mind that he’s just read that I can’t have penetration, he just shoves, and that is the only word that I can describe, his hand into, like into my vagina, and I screamed and almost fell off the bed, and… where was the compassion? Where was the consideration?…so I’ve got, I’ve just got no trust in doctors, I, I’m angry, I just, I just, I hate them (Laura).

This extract can be seen as illustrative of the power of the clinician and Laura’s vulnerability. Overall, this left the lead researcher with a sense of assault and violation, for several reasons. Firstly, the phrase “I want to examine you” is a statement, not a question, and as such cannot be aimed at seeking consent for examination from the patient. The definition of consent is “to gain permission for something to happen or agreement to do something” (http://www.en.oxforddictionaries.com). The urologist does the opposite, according to the participant: “without warning me, bearing in mind that he’s just read that I can’t have penetration, he just shoves, and that is the only word that I can describe, his hand into, like into my vagina.” At no point does the participant describe the urologist asking whether he can put his hand in her vagina. She describes being unaware of any reason why he would “need to do anything with my vagina.” If she is unaware he is going to insert his hand into her vagina, then he has not explained that this is what he will be doing, and why this may be necessary; therefore, he had not obtained informed consent.

Secondly, Laura is not given information about the purpose of the examination; instead she describes the lack of compassion and consideration (both traits associated with being treated “humanely”: “Having or showing compassion or benevolence” [http://www.en.oxforddictionaries.com]). To the researcher, it felt as though Laura experienced being treated like an object to be examined, rather than a human patient worthy of compassion and consideration. The use of the words “shoved” and “screamed” within the same sentence produces a sense of the incident as violent, painful, and abusive. Thus, the experience feels non-consensual and objectifying, and leaves Laura, unsurprisingly, with “no trust in doctors” and feelings of “anger” and “hatred,” and yet still reliant on remaining in the healthcare system to move forward.

In contrast to this experience, other participants viewed examinations as positive and a necessary part of the journey toward diagnosis (Liz, Bessie, Vicky).

Well I took it as par for the course; because it’s necessary…I was glad to have an examination (Liz).

Overall, women’s encounters with some professionals who prioritized the maintenance of their power and keeping patients as passive (“patient”), led five of the eight women to speak of their desire to complain (Lilly, Amy, Vicky, Laura and Liz). Despite this, only Liz wrote to Patient Advice and Liaison Service (PALS) to make a complaint, but she did not wish to pursue it. The women cited fear of their future care being compromised as the reason why they did not complain, believing a need to keep on the (good) side of health professionals and administrative staff alike, in order for their care to continue unharmed, thus further illustrating the perceived power held by the healthcare system.

### Sub-theme 3: The Psychosocial Impact of the Journey

Nearly all the participants described a variety of psychological consequences, which they associated with the process of journeying through the healthcare system, including: “isolation,” “panic,” “depression,” “anxiety,” “low self-esteem,” “hopelessness,” “rage,” “fear,” and “humiliation” (Clara, Josephine, Laura, Amy, Liz, Lilly, Vicky). All of these impacted on sex and relationship difficulties (Laura; Lilly), work/education (Bessie; Liz; Amy; Clara), and sleep difficulties (Vicky; Josephine). Moreover, some women recognized the impact of psychological triggers exacerbating pain, connecting “stress” with “flare ups” (Lilly).

Despite recognizing the psychological triggers and consequences of living with vulvodynia, the women described feeling angry at suggestions that the pain was “all in my head/mind” (Liz, Bessie, Laura, Vicky, Josephine). Women did not identify with health professionals’ suggestion that vulvodynia had a psychological cause, instead perceiving this as another barrier on their journey. Thus, some women became “desperate” (Amy) to find a cause with a physical biomarker. The importance of having a concrete and physical cause was so great that Lilly resorted to surgery (against medical advice) in an attempt to externalize and remove the pain from herself. In this extract, Lilly laughs throughout the description of something that she describes as “awful,” suggesting that Lilly is defending against the pain arising from not being able to find a physical cause.

I thought all the pain was stemming from the fact that maybe there was something erm [laughs] like physically wrong with my labia, so I thought maybe I should get my labia removed [laughs] God, it’s so awful’… ‘so actually when I was [laughs] when I was erm, not [sighs] when I was 32 I did have labiaplasty (mmm), cause I was just so convinced that was why I was in pain (mmm) and actually it hasn’t helped, it hasn’t made any difference whatsoever, [laughs] (Lilly).

Women’s consistent non-acceptance of a psychological cause—in the context of their acknowledgment that their pain was exacerbated by psychological triggers, and their recognition that being in the healthcare system had had psychological consequences—suggests that the notion of a psychological cause for their pain did not make sense for them.

Finally, the experience of persisting on the journey within the healthcare system became too much for Lilly: “I don’t, honestly don’t have the energy for it,” and she removed herself from the system by no longer engaging with it, in a similar way to those who sought private healthcare. This halting of the journey demonstrates that for Lilly, the experience of continuing to live with chronic vulval pain was preferable to remaining within a perceived iatrogenic healthcare system.

### Master Theme 2: “What is Vulvodynia?”: Ambivalence Toward Diagnosis

Some participants felt very strongly that diagnosis was the key to moving forward (Amy; Laura), while others considered diagnosis to be just a name (Vicky; Bessie; Lilly), with little or no validity, reliability, or predictability. Others showed some uncertainty about the role of diagnosis, acknowledging both its helpfulness and its hindrance (Clara; Vicky; Liz; Josephine). Thus, there was an overall sense of group ambivalence toward diagnosis. The following two sub-themes demonstrate the polarization of views on diagnosis, as well as the ambivalence both within and between individuals:

### Sub-theme 1: “Vulvodynia is a Bit of a Cop-Out” (Clara): Limitations of a Diagnosis

Six participants raised concerns regarding the limitations of the diagnosis (Vicky, Bessie, Lilly, Clara and Liz). First and foremost, some participants were unsure what the term vulvodynia actually meant, feeling that vulvodynia was “only a name” (Bessie) which “just means vulval pain” (Clara) and as such, was unable to explain the pain or predict “a cure” (Clara). Some women felt that health professionals told them it was vulvodynia “because they don’t know what it is” (Bessie), perhaps suggesting that women’s experience of receiving a diagnosis was that it served a purpose, perhaps to prevent doctors from having to say “I don’t know,” or as a way of maintaining the idea of medicine as a panacea. Further, some women felt that they had been misled by professionals, whom they had experienced as giving the diagnosis as though it had explanatory and predictive power, only to later find out that it did not.

Well I think it’s, if, if they’d have said, “It is only a name and it just means a pain,” but it was as if “oh, it’s vulvodynia,” and you think “oh, got a name, vulvodynia,” and then you realize it, it’s not going to help anything cause there’s no answer to it (Bessie).

Women also described a sense of losing faith in the diagnosis:

It’s hard to believe that that’s what you’ve got, when what they’re given you isn’t making it any better, but, however you want the treatment to start making an impact…or else you start doubting that diagnosis…(pause) that tells you something doesn’t it? When you’ve tried everything (Liz).

Liz uses a rhetorical question “that tells you something doesn’t it?”, forcing the listener/reader to think more about the limitations of the diagnosis. Both Liz and Bessie’s quotes illustrate their expectations of what a diagnosis should provide. Firstly, Bessie had hopes and expectations that receiving a diagnosis of vulvodynia would provide “answers” as to the cause of the pain. Secondly, Liz expected that a diagnosis should predict an effective treatment. The term “vulvodynia” failed on both counts for both women. Both women believed the notion of the medical model as a panacea, and both began to realize their expectations were not going to be fulfilled by a traditional medical model approach of examine, diagnose, treat, and cure.

For Vicky and Clara, the need for a “name” lessened throughout the course of the journey. Instead, these women emphasized the need to be treated as a “whole person and not just a set of symptoms” (Clara) and for sense-making over labeling:

Having a name doesn’t matter, having some (pause) I think having a kind of narrative about what’s gone on what it’s all about what’s, what’s the story of it ‘cause I think people do need to make sense of what’s going on (Vicky).

Overall, some participants felt that a diagnosis of vulvodynia did not offer validity, reliability, or predictability, making it useless and leaving them to speculate that the purpose of a diagnosis was more for the benefit of health professionals.

### Sub-theme 2: The Value of Diagnosis

Despite these reservations, some participants saw the benefit in a diagnosis; providing “relief” (Clara, Laura and Amy), confirming a sense of what they had “known all along” (Clara), allowing professionals to communicate that they had “a grasp of it” (Clara), providing peace of mind that there was not something “seriously wrong” (Liz), “putting some sense” to how they were (Liz), and facilitating professionals to “point me in [the right direction]” (Laura).

I’ve got pain, you know, at least, if you can call it something, it’s kind of given me the confidence to tell people…But I have, in the last, only in the last six months or so, bearing in mind I’ve lived with this for years and years, erm, told my close friends about it, and they’ve, they’ve been absolutely amazing, much better than I ever imagined them to be, to be honest and I think this is all contributing to me feeling a lot better, and being in a better place (Laura).

As Laura’s quote illustrates, some women also found that a diagnosis afforded them the vocabulary to facilitate communication with their family and friends about their pain, in turn allowing them to test out their fears that rejection and stigma would follow if they disclosed their secret. When these fears were not realized following disclosure, women felt less isolated and more supported by significant others in their experience of the pain (Laura, Amy). In particular, both Laura and Amy conveyed a sense of having a diagnosis as alleviating blame which was paramount to their journey. Interestingly, both women (who were in their early twenties) were repeatedly referred to sexual health clinics, which they experienced as stigmatizing. Their sense of relief at receiving a diagnosis might suggest that, rather than actively providing anything meaningful (an explanation or effective treatment), a diagnosis was important to them because it removed a sense of stigma and shame. This was perhaps felt most acutely by young women fearful of being labeled as “sexually promiscuous” (a label deemed to be more negative for women than men (Crawford & Popp, 2003)), due to repeated referrals to the sexual health clinic: “No more STI clinic for me” (Amy).

Furthermore, Vicky noted the importance of diagnosis in gaining “respect,” perhaps also commenting on the ability of a diagnosis to remove any stigma surrounding pain and sex and as such provide relief:

Maybe that is the difference between being respected and not, to a doctor giving it that name is the difference between a patient being respected and a patient not being respected (Vicky).

### Master Theme 3: Patriarchy, Women, and Sex

I’d like to know where it’s [the term “vulvodynia”] come from, whose put that name to…I bet it’s a man (Bessie)

The women often used words and phrases suggesting that they had encountered patriarchal attitudes along their journey. Bessie believed the term vulvodynia had been thought up by “a man”; her quote suggests that for her, the very name “vulvodynia” may embody a sense of men controlling her experience by creating a name that fails to adequately capture her experience and expectations. The following three sub-themes outline the women’s experiences of the medical profession as entrenched with patriarchal attitudes.

### Sub-theme 1: Narratives Around Womanhood: A Barrier on The Journey

Women experienced suggestions from medical professionals that they were “neurotic” (Clara and Vicky), “mithering” (Liz), “pestering” (Liz; Amy), and/or “hysterical” (Vicky). Such language, often specifically directed toward women (Romaine, 2000), communicates a sense that the women often felt dismissed and that their concerns, distress, and experiences were belittled and considered unimportant. Further, Lilly and Vicky described receiving advice that was experienced as patronizing, such as “have a glass of wine and get into the bath,” and gendered, such as “do things like knitting, to take your mind off it,” which communicated a sense that professionals were not understanding or taking their pain seriously. Clara felt that this lack of understanding and patronizing treatment was down to her gender:

A lot of doctors just (pause) perhaps they don’t even realise it but perhaps they have these preconceptions about females and even female doctors, erm, and they, it affects how they are with women and I, I can’t speak for all women, but, it was strange that when I’ve talked with other women, they’ve kind of said yeah I’ve had that kind of experience (Clara).

Clara states she feels “fobbed off” (“Deceitful attempt to satisfy someone by making excuses or giving them something inferior.” http://www.en.oxforddictionaries.com) by doctors, believing that the perception she thinks doctors have of women—that they make “mountains out of molehills”—(Clara) leads them to dismiss women’s concerns as not serious. She directly links this feeling of not being taken seriously to being a woman, recounting how the “other women” she has talked to have also had “that kind of experience.” Thus, Clara believes that some women are routinely treated differently to their male counterparts by doctors, simply because they are women.

The women recounted difficulties asserting themselves, perhaps for fear of being labeled as “neurotic,” or perhaps due to feeling repeatedly being dismissed. They described the need to be obedient (“Whatever anybody tells me to do, I do it” (Liz)), not challenging incorrect hypotheses of doctors (“But I didn’t want to upset him [consultant] by telling him it wasn’t my cough cause he was delighted with that” (Bessie)), and continuing to be “nice” (Liz).

### Sub-theme 2: Female Sexuality, Shame, and Stigma

Having sex is either for the benefit of men or, or for having babies and (hmm), and and it’s erm a little bit unseemly for women to openly admit that they might just do it for the fun of it (Vicky)

By its very nature, vulvodynia was associated with sex. This quote from Vicky illustrates her sense of the unimportance assigned to female sexuality for women within the healthcare system. This feeling was echoed by Laura and Lilly and suggests the women experienced a prevailing belief within the healthcare system that the purpose of women’s sexuality is to function only as a “performance” (Laura) for men, and not to be for the purpose of their own enjoyment. In essence, it seemed as if women believed that, within the healthcare system, they were viewed only as baby-makers or pleasure-givers, removing a sense of women’s ownership over their sexuality. This was in contrast to how women perceived men’s sexuality was viewed by the healthcare system:

What I honestly feel about this is, erm, if I was a guy, and I was saying, like I can’t get erections, I’m not able to ejaculate (mmm) something like that (hmm mmm) then it would be taken really seriously (Lilly).

Moreover, women reported a sense of stigma associated with sex, which served to delay progression of the journey. While all the participants talked about stigma and shame attached to experiencing pain, the pre-menopausal women seemed much more vulnerable to experiencing shame and stigmatization through being referred, often repeatedly, to sexual health clinics. These services were consistently associated with shame and stigma by all the women who visited them (Vicky, Amy, Laura).

Being just sent down and in actual fact I waited four I just had to go down and wait four hours (hmm), walk in off the street and wait for four hours and so that in itself I think was disrespectful (hmm), to instead of referring [02.04] erm and it’s it’s a pretty horrible place (hmm), it’s a pretty horrible place and erm it’s a nice building but the whole process of it is quite dehumanizing (Vicky).

Vicky’s quote above alludes to shame and stigma, while Laura referred to being made to feel “dirty,” and Amy worried about being labeled as a “trollop” or a “skank.” This quote is suggestive of powerful negative narratives around female sexual health and their potential impact upon women’s sense of feeling shamed. Interestingly, Vicky, who identified as a feminist and was able to analyze her experience within a feminist framework, was critical of the system, which she felt had been specifically set up to create shame around sex. Amy and Laura, on the other hand, were not critical of the system, but rather distanced themselves from the stigma attached to sexual health by using language to stigmatize others to create a “self” and “other” narrative, with “self” referring to somebody who is “clean as a whistle” (Amy) and “other” referring to women who have sexual health problems viewed as “vulgar” and “green” (Amy). Moreover, both Amy and Laura use the phrase “I’m not like that” in order to distance themselves from the perceived negative labels attached to women attending sexual health centers. This experienced stigmatization of women’s sexuality is highly influential on a journey on which women report feeling judged, stigmatized, and shamed, all of which has potential implications for the well-being and mental health of women with vulvodynia (see Master Theme 1, Sub-theme 3: The Psychosocial Impact of the Journey).

### Sub-theme 3: “The Old Boys’ Brigade” and The Medical Profession

All women described instances whereby they had experienced patriarchal attitudes in the system, such as being patronized and dismissed, treated without dignity or compassion, or having their difficulties belittled and instead attributed to female “neuroticism.” However, Vicky was particularly eloquent and passionate about her views on why women encounter such experiences, supported also by the views of Josephine (“the old boys’ brigade”) and the encounters described by the other women.

Vicky explicitly described her experiences as “misogynistic”:

It’s about the essential misogyny of the health profession, I’m afraid…the medical profession, it’s about…you know this profession that’s erm (pause) even though women are in it, incredibly male dominated and hierarchical and erm you know dominated by people from public schools and so it it it’s erm (pause) it’s a profession that’s full of very old idea and I think women come on the receiving end of that all the time I think doctors frequently, male doctors especially, frequently patronize women (hmm hmm), erm and treat women’s problems as…lesser (Vicky).

Clara described her surprise that she encountered “preconceptions about females” from both male and female doctors, and Josephine compared her experiences with medical professionals to other systems including “politics” and “religion,” describing the medical profession as “the old boys’ brigade.” The notion of women’s difficulties being treated as “lesser” was further supported by Lilly, who felt that “women’s health isn’t taken seriously at all” (Lilly). Vicky in particular had an interpretation of her experiences which encompassed a general, collective view of women as unimportant, because they are not seen as valuable contributors in a society where importance is based upon economic value:

erm…I think [sighs] I think it’s terms of of of of kind of NHS priorities I think the fact that erm vulval pain is something that is suffered by people who are often not a big part of the earnings makes it a low economic priority in in terms of kind of research and treatment you know we don’t, a lot of the women concerned are sort of middle aged, maybe not working (Vicky).

Throughout this extract, Vicky talks about “earnings” and “economic priority,” suggesting that she believes her experiences within the healthcare system may have been influenced by a cost–benefit analysis of treating women with vulvodynia. She is suggesting that care within the healthcare system is influenced by notions of patient importance and that importance is based upon economic earning power, which is generally less for women than for men, for a variety of reasons (e.g., pay gap inequality; larger percentage of women engaged in unpaid caring and domestic work). As such, Vicky implies that there is inequality within the healthcare system, to the detriment of women.

The notion of a “misogynistic” healthcare system may go some way to explain the experience of women described in Master Theme 1, Sub-theme 2: The status and power of the medical model, such as being violated, silenced, and dismissed during their journey.

## Discussion

To fill a gap in our understanding of why so few women experiencing symptoms of vulvodynia seek help, and why even fewer obtain a diagnosis (Reed et al., 2012), the current study asked women about their journey within the UK healthcare system toward obtaining a diagnosis of vulvodynia.

The women interviewed reported lack of knowledge by medical professionals (particularly GPs) about vulvodynia to be a barrier on their journey. This is of particular importance when GPs act as a gateway to other more specialist services. In the current study, women’s perceptions of doctors’ lack of knowledge are partly supported by quantitative data regarding the knowledge of junior doctors about vulvodynia. Toeima and Nieto (2011) found that more than 60% of junior doctors in the UK underestimated the prevalence of vulvodynia, more than 80% had never attended an educational session or training course on vulvodynia, and more than 70% were not aware of the new ISSVD classification (Moyal-Barracco & Lynch, 2004). Further, Toeima and Nieto note that despite dedicated vulval pain clinics in the UK, women were frequently referred by their GP to general gynecology clinics. As well as medical professionals’ limited knowledge of vulvodynia, some women also described how they felt belittled, dismissed, and violated following encounters with them, which is echoed by a previous study into the experiences of women with a variety of chronic pain disorders (Werner, Isaksen, & Malterud, 2004), as well as previous feminist sexology literature (Martin, 1987) which documents how women’s problems are often viewed as inherently due to their own failings, leading professionals to negate the needs and concerns of women (Gannon, 1998). Interestingly, previous research has reported that in women with chronic pelvic pain, favorable assessment of the medical consultant by the patient in the first consultation predicts resolution of pain (Selfe, Matthews, & Stones, 1998). Selfe et al. suggest that the consultation style of individual doctors may be important, especially in the context of “chronic and ill-defined conditions, where patients are distressed and hostile and an immediate curative intervention is elusive” (p. 1047).

The suggestion that women’s pain was “all in their head” as reported by the women interviewed in the current study has previously been described in the wider sexology literature, especially relating to a bind that women find themselves in in relation to the experience of PMT (Martin, 1987), and also in more specific research relating to the experience of vulvodynia (Brotto et al., 2013; Marriott & Thompson, 2008; Sadownik et al., 2012b). Marriott and Thompson (2008) propose that women felt that a “medical condition” could be externalized and hopefully removed, but that a psychological aspect of their pain indicated something wrong internally “in a core aspect of themselves” (p. 252). In contrast, the current study found that women were open to psychological aspects of their pain, but strongly resisted the notion that their pain had a psychological cause, which did not make sense to them, and therefore was not felt to be accurate. Both Marriott and Thompson’s (2008) findings and those of the current study suggest that caution should be exercised when communicating with women about psychological aspects of pain. Clear distinctions must be made between psychological triggers (e.g., stress events, bereavement, pressure), which may exacerbate pain; psychological consequences (low mood, anxiety, anger) which may occur due to the experience of the pain or to encounters within the healthcare system; and psychological causes (trauma, abuse, neglect) of vulvodynia, a notion that was rejected by the women in the current study as not relevant to them, serving only as a barrier on their journey to understanding and treating their pain.

While no studies have specifically examined women’s views on the usefulness of the term “vulvodynia,” previous literature has highlighted the ambivalence associated with other diagnoses, such as “fibromyalgia” where the diagnosis may be poorly defined and/or difficult to diagnose (Dennis, Larkin, & Derbyshire, 2013). In the current study, some women found the term helpful (especially in gaining “respect” from doctors), but there was also a sense from others that it was “just a name,” and therefore of limited benefit. Indeed, the complexity of other idiopathic disorders, such as chronic pelvic pain, has led some to suggest that exhaustive pursuit of a precise diagnostic label may not necessarily be productive (Selfe et al., 1998). Thus, the benefits and limitations of the label “vulvodynia” should be acknowledged and discussed with women when the diagnosis is given, in order to avoid a false impression that the diagnosis of vulvodynia leads to immediate and curative treatment. Moreover, it is important to recognize that the “journey toward diagnosis” is only part of the journey, and as noted by the women interviewed here, does not necessarily secure appropriate care and effective treatment. Indeed, future research should focus upon what women have found most helpful following diagnosis.

In terms of these women experiencing the healthcare system as “misogynistic,” previous literature suggests that doctors may sometimes perceive female patients as “inherently dependent” and “lacking in common sense,” a view that rationalizes “paternalistic attitudes and advice” (Gannon 1998, p. 295), which may help to explain why some women in the current study experienced being given “patronizing advice” from doctors. This is in keeping with the previous literature which highlights clinicians’ often androcentric views of sexuality (Du Plessis, 2015; Maines, 1999), suggesting that physicians may instruct female patients on values, morals, and sexual behavior (Gannon, 1998), which is further supported by the experiences of participants in the current study. Many of the women reported experiencing shame and stigma surrounding sexual health in the context of the healthcare system and, in some cases, suggestions that their sexuality was unseemly, or only for the purpose of child-bearing, or the pleasure of men, demonstrating that consistent with the feminist literature of the 1990s (Krieger & Fee, 1994), women’s needs continue to be viewed in the context of maternal and child health, ignoring the other multifaceted and individualized needs of women. Finally, the concern of women in the present study that they would be labeled as “neurotic” or “hysterical” (labels historically reserved solely for women (Tasca et al., 2012)), illustrates the detrimental and silencing impact that these labels still have on women today.

The current study supports previous literature which has outlined the psychological difficulties experienced by some women with vulvodynia (Buchan et al., 2007; Kaler, 2006; Marriott & Thompson, 2008; Sadownik et al., 2012b). Moreover, this study suggests that encounters with the healthcare system during the journey toward diagnosis may be experienced as actively harmful to women’s psychological wellbeing, resulting in psychosexual difficulties, anxiety, and depression. This may help to explain why only around half of all women experiencing symptoms consistent with vulvodynia seek treatment (Connor et al., 2013).

### Implications

More research is needed to explore the issue of vulvodynia which affects up to 16% of women. Such research should address gaps in our knowledge, including the issues raised by previous feminist literature, with findings to be translated into clinical practice through education and training. Psychologists can offer one-to-one and/or group work (where appropriate) for women who have suffered in their journey toward diagnosis, addressing secondary sexual difficulties, such as vaginismus, and other psychological problems such as anxiety and depression, and feelings of shame and stigma. Clinical and health psychologists could also provide education, training, supervision, and consultation for medical and other healthcare professionals, highlighting issues such as the impact of the power imbalance between professionals and women with vulvodynia, and how this may act as a barrier to treatment. Other areas of possible training include (1) the psychological impact of vulvovaginal examinations and their potential to be experienced as an assault, unless done sensitively and with informed consent; (2) the psychological impact of either inappropriate referrals, delays in referrals to specialist vulval clinics, or repeated referrals to avenues already explored (such as sexual health clinics); and (3) education about the nature of vulvodynia and the differences between psychological causes, triggers and consequences, and how to discuss these with women.

### Methodological Considerations

The current study included both pre- and post-menopausal women, which may be deemed to breach the homogeneity needed for the conduct of IPA research. However, the decision to exclude women based on pre- or post-menopausal status in previous research (Marriott & Thompson, 2008; Sadownik et al., 2012a, b) was viewed as unhelpful, given that both groups are affected by vulvodynia and within this small sample both recounted similar experiences in their journeys toward diagnosis. In addition, seven women had a co-morbid diagnosis, contributing to the complexity of their case and potentially further hindering their journeys toward a diagnosis of vulvodynia. However, the co-morbidities presented here are reflective of a population of vulvodynia patients (Arnold, Bachmann, Kelly, Rosen, & Rhoads, 2006). Finally, as with all previously identified studies examining women’s subjective experiences of vulvodynia (Ayling & Ussher, 2008; Brotto et al. 2013; Buchan et al., 2007; Kaler, 2006; Marriott & Thompson, 2008; Munday et al., 2007; Sadownik et al., 2012a, b), the current study includes only white, British women in heterosexual relationships. Future research should aim to explore the experiences of lesbians, bisexual women, trans people, and women from diverse ethnic backgrounds. Moreover, it could be argued that women who have had negative experiences with the healthcare profession may be more likely to volunteer to participate in qualitative research, creating a further bias.

Despite these limitations, the current study has several strengths, including consultation throughout the research process with members of the VPS, researching an issue that was deemed to be important by women, use of empirical guidelines for collection and analysis of data, and an adequate sample size for the purpose of an IPA study.

### Future Directions

Future research could focus on women’s experiences of specialist vulval pain clinics (as well as the effectiveness of such services); what initiatives can be put in place to ensure women feel able to seek and access services (including the training of clinicians in vulvodynia and the gendered and social influences on the experience of its symptoms); the experience of healthcare following diagnosis; and the benefits of psychological approaches in improving women’s mental health along the journey. Research could explore the outcomes linked to training health professionals regarding the psychological aspects of vulvodynia, and the perceived impact of this upon women’s experiences and satisfaction with their subsequent consultations.

### Conclusion

The current research offers an in-depth insight into women’s experiences of their journey toward a diagnosis of vulvodynia, highlighting the difficulties associated with this process. This may go some way to explaining why so few women obtain a diagnosis of vulvodynia, despite the experience of symptoms consistent with it. Further, diagnosis is not the end of the journey for women with vulvodynia, nor is it predictive of a cure or even effective management; instead, diagnosis is only part of women’s experience. Future research and clinical practice should focus on how to promote a healthcare system conducive to women successfully being given help for symptoms, above and beyond a diagnosis.

## Footnotes

1 Phenomenology is the philosophical study of the structures of subjective experience and consciousness. Hermeneutics is the theory of interpretation. Idiography is the effort to understand the meaning of contingent, unique and often subjective phenomena of individuals.
